# Supplementary material for: Development and Evaluation of an AxiomTM 60K SNP Array for Almond (Prunus dulcis)
Source: Plants (Basel). 2023 Jan 5;12(2):242. doi: 10.3390/plants12020242 (PMC9866729; doi:10.3390/plants12020242)

Supporting information

Table S1: Summary of the minimum, maximum and average number of SNP over a window of one million base pairs for each chromosome, array data.

Chr	Min	Max	Mean
1	75	506	284
2	51	799	297
3	22	490	274
4	30	468	249
5	50	460	300
6	97	491	272
7	47	637	300
8	113	587	310

Table S2 : List of the twenty almond varieties scored "BB" for the SNP AX-599403227 liked to the bitter taste of the kernel and their phenotype for sweetness.

**code INRAE**	**NOM_VAR**	**Country**	**AX-599403227**	**Phenotype**
R881	Atocha	ESP	BB	sweet
R163	Mollare de Tarragona	ESP	BB	sweet
RE37	Pestaneta	ESP	BB	sweet
RE39	Rumbeta	ESP	BB	sweet
R1025	A la Dame	FRA	BB	sweet
R961	Aveyron	FRA	BB	sweet
R1443	Dame Franche	FRA	BB	sweet
R192	Flots	FRA	BB	sweet
R1440	Fascionello	ITA	BB	sweet
R530	Fragiulio	ITA	BB	sweet
R596	Dreik Nikitsi	SUN	BB	sweet
R762	Nikitsi N°17	SUN	BB	sweet
R697	Northland	USA	BB	sweet
R270	Texas	USA	BB	sweet
R966	P. dehiscens x dulcis	FRA	BB	bitter
R1086	P.bucharica x peach	FRA	BB	bitter
R1107	ALNEM-1	ISR	BB	bitter
R1109	ALNEM-201	ISR	BB	bitter
R992	PG AMMAN	JOR	BB	bitter

Table S3: Detailed sample information for resequenced accession.

Species	Sample_ID	Accession	Country	Sequence	Reference
P dulcis	R691	Chellaston	Australia	CNAG_CRG	this study
P dulcis	RA1	Johnston´s prolific	Australia	CNAG_CRG	this study
P dulcis	RA2	Keanes	Australia	CNAG_CRG	this study
P dulcis	RA3	McKinlays	Australia	CNAG_CRG	this study
P dulcis	RA4	R23T45	Australia	CNAG_CRG	this study
P dulcis	RA5	Strouts papershell	Australia	CNAG_CRG	this study
P dulcis	RA6	UA03	Australia	CNAG_CRG	this study
P dulcis	RA7	UA05	Australia	CNAG_CRG	this study
P dulcis	DL08	A Yue Hun Zi Xinjiang	China	NCBI	Yu et al 2018
P dulcis	DL05	Ai Feng Xinjiang	China	NCBI	Yu et al 2018
P dulcis	DL06	Ba Dan Wang Xinjiang	China	NCBI	Yu et al 2018
P dulcis	DL12	Bian Zui He Xinjiang	China	NCBI	Yu et al 2018
P dulcis	DL10	Da Ba Dan Xinjiang	China	NCBI	Yu et al 2018
P dulcis	PD09	DPRU 2331.9	China	NCBI	Velasco et al 2016
P dulcis	DL03	Gong Ba Dan Xinjiang	China	NCBI	Yu et al 2018
P dulcis	DL07	Huang Shuang Xinjiang	China	NCBI	Yu et al 2018
P dulcis	DL09	Tao Ba Dan Xinjiang	China	NCBI	Yu et al 2018
P dulcis	DL04	Wan Feng Xinjiang	China	NCBI	Yu et al 2018
P dulcis	DL11	Ye Er Qiang Xinjiang	China	NCBI	Yu et al 2018
P dulcis	DL02	Zhi Pi Xinjiang	China	NCBI	Yu et al 2018
P dulcis	R1025	A la Dame	France	CNAG_CRG	this study
P dulcis	R269	AI	France	CNAG_CRG	this study
P dulcis	R61	ARDECHOISE	France	CNAG_CRG	this study
P dulcis	R164	BARTRE	France	CNAG_CRG	this study
P dulcis	R216	BELLE D'AURONS	France	CNAG_CRG	this study
P dulcis	R1028	Beraude	France	MGX	this study
P dulcis	R142	Cote d'or	France	MGX	this study
P dulcis	R197	Dorée	France	CNAG_CRG	this study
P dulcis	PD10	DPRU0210,LANGUEDOC	France	NCBI	Velasco et al 2016
P dulcis	R486	Ferragnès	France	CNAG_CRG	this study
P dulcis	R800	Ferrastar	France	CNAG_CRG	this study
P dulcis	R916	Lauranne	France	MGX	Van Ghelder, al 2019
P dulcis	R190	Pointue d'Aureille	France	CNAG_CRG	this study
P dulcis	R1046	Princesse-JR	France	CNAG_CRG	this study
P dulcis	R244	Saint charles	France	MGX	this study
P dulcis	R195	Tardive de la Verdière	France	MGX	this study
P dulcis	R692	RETSOU	Greece	CNAG_CRG	this study
P dulcis	PD04	DPRU 2374.12	Iran	NCBI	Velasco et al 2016
P dulcis	R1107	Alnem	Israel	MGX	Van Ghelder, al 2019
P dulcis	R210	CRISTOMORTO	Italy	CNAG_CRG	this study

P dulcis	PD06	DPRU 2301, TUONO	Italy	NCBI	Velasco et al 2016
P dulcis	R1099	Falsa Barese	Italy	CNAG_CRG	this study
P dulcis	R856	Genco	Italy	CNAG_CRG	this study
P dulcis	PD05	DPRU 1456.4 BADAM	Pakistan	NCBI	Velasco et al 2016
P dulcis	PD07	DPRU 1462.2	Pakistan	NCBI	Velasco et al 2016
P dulcis	R881	Atocha	Spain	CNAG_CRG	this study
P dulcis	R1575	Atocha_M	Spain	MGX	this study
P dulcis	R1549	Belona	Spain	CNAG_CRG	this study
P dulcis	*PD12*	*D05-187*	Spain	NCBI	Koepke et al 2013
P dulcis	R1498	Del Cid	Spain	MGX	this study
P dulcis	R308	Desmayo.LARGUETA	Spain	CNAG_CRG	this study
P dulcis	R1197	GABAIS	Spain	CNAG_CRG	this study
P dulcis	R981	Garfi	Spain	CNAG_CRG	this study
P dulcis	R1576	Garrigues_M	Spain	MGX	this study
P dulcis	R934	GUARA	Spain	CNAG_CRG	this study
P dulcis	R185	MARCONA	Spain	CNAG_CRG	this study
P dulcis	R1546	MARINADA	Spain	CNAG_CRG	this study
P dulcis	R1514	Peraleja	Spain	MGX	this study
P dulcis	*PD14*	*RAMILLETE*	Spain	NCBI	Koepke et al 2013
P dulcis	R1427	Ramillete_M	Spain	MGX	this study
P dulcis	*PD11*	*S3067*	Spain	NCBI	Koepke et al 2013
P dulcis	R1547	VAIRO	Spain	CNAG_CRG	this study
P dulcis	R1574	VIALFAS	Spain	CNAG_CRG	this study
P dulcis	R1529	VIVOT	Spain	CNAG_CRG	this study
P dulcis	R726	Achaak	Tunisia	CNAG_CRG	this study
P dulcis	PD03	DPRU 1791.3, BE-1609	Turkey	NCBI	Velasco et al 2016
P dulcis	PD01	DRPU 2578, #53	Ukraine	NCBI	Velasco et al 2016
P dulcis	R613	PRIMORSKI	Ukraine	CNAG_CRG	this study
P dulcis	DM13	Ao 2	USA	NCBI	Yu et al 2018
P dulcis	DM15	Mission	USA	NCBI	Yu et al 2018
P dulcis	R1577	Mono	USA	MGX	this study
P dulcis	DM14	Nonpareil	USA	NCBI	Yu et al 2018
P dulcis	R707	Ripon	USA	CNAG_CRG	this study
P dulcis	R270	Texas	USA	GDR	Alioto et al 2019
P dulcis	R1088	Titan	USA	MGX	this study
P dulcis	PD08	DPRU 1207.2	Uzbekistan	NCBI	Velasco et al 2016
P webbii	R755	A. webbii	Bulgary	CNAG_CRG	this study
P.fenz.x P.buch.	R771	P.fenzl x P.bucharica-2	France	CNAG_CRG	this study
P bucharica	R1553	P bucharica	Spain	CNAG_CRG	this study
P kuramica	R1552	P kuramica	Spain	CNAG_CRG	this study
P webbi	R1578	P. webbi_M	Spain	MGX	this study

Figure S1: Cluster plots of the Three additional SNP

A) AX-599403222

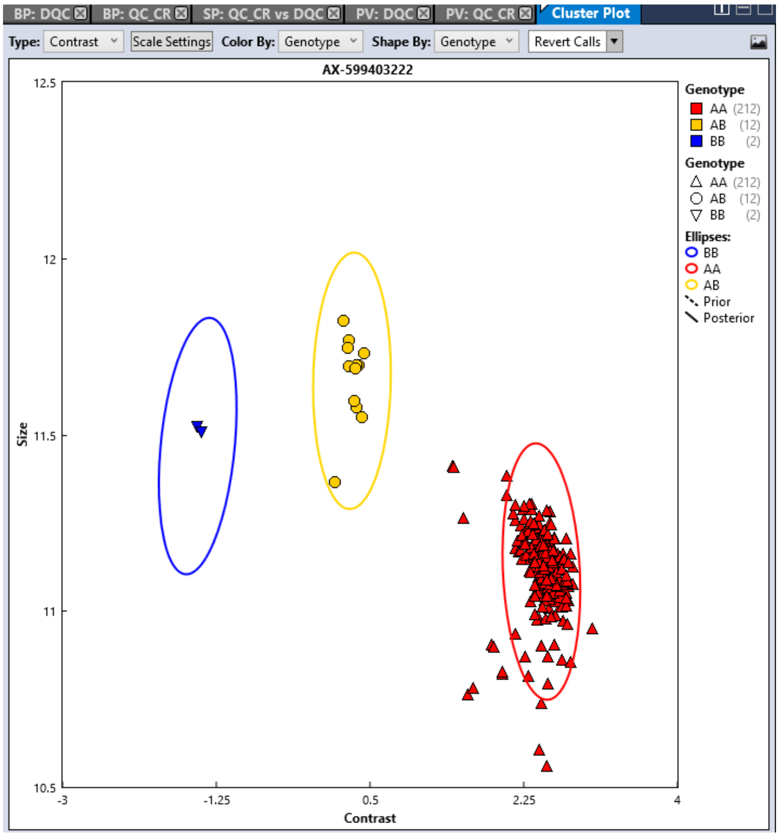

B) AX-599403226

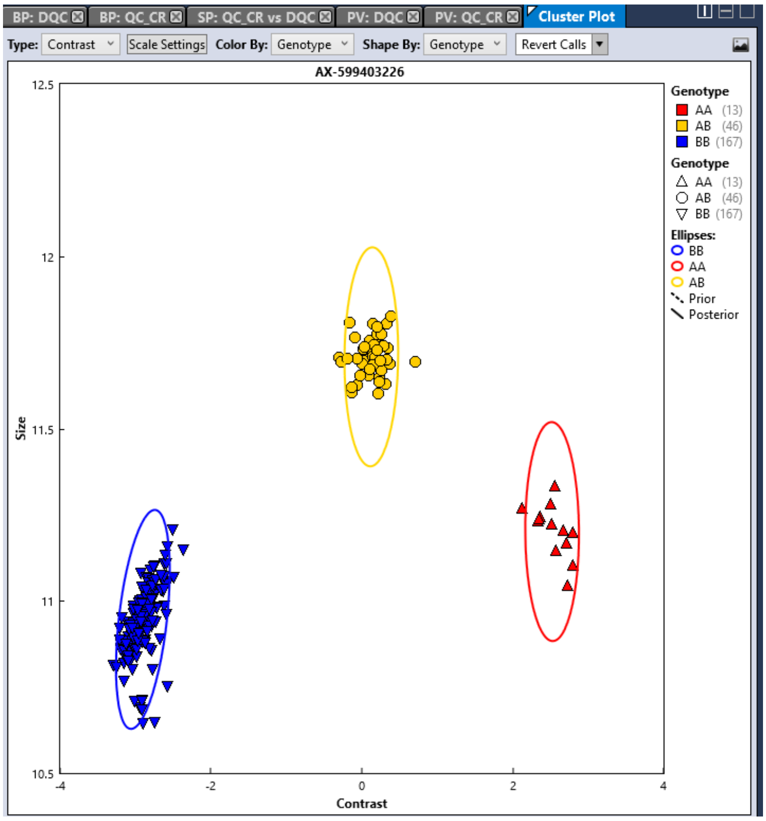

C) AX-599403227

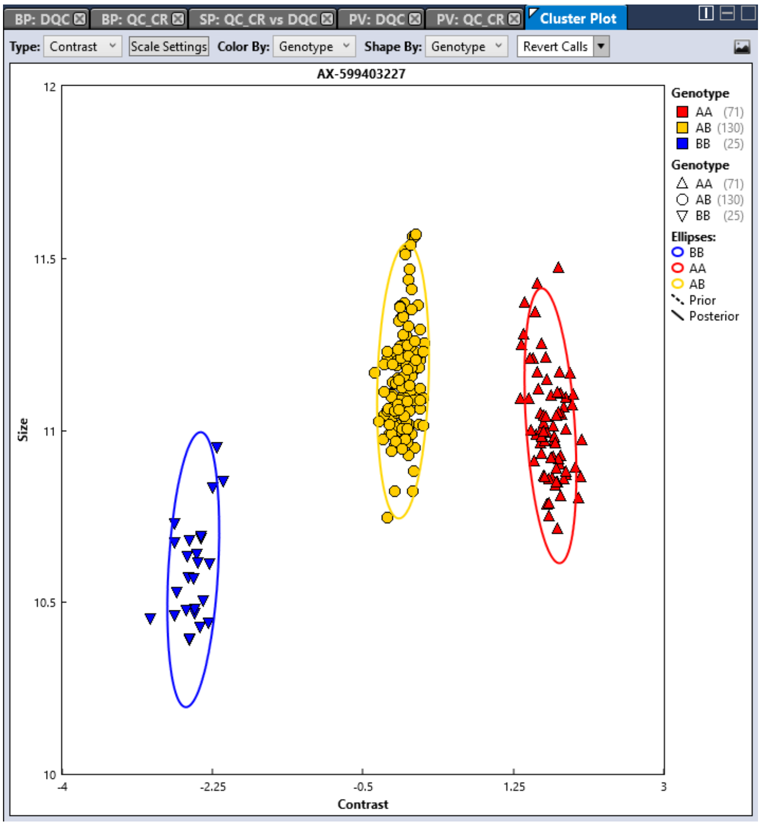

Supplement: Supplementary file 1 [file plants-12-00242-s001.zip › plants-2041375-supplementary.pdf]
